# Design and Development of a Novel Peptide for Treating Intestinal Inflammation

**DOI:** 10.3389/fimmu.2019.01841

**Published:** 2019-08-06

**Authors:** Lulu Zhang, Xubiao Wei, Rijun Zhang, Jim N. Petitte, Dayong Si, Zhongxuan Li, Junhao Cheng, Mengsi Du

**Affiliations:** ^1^Laboratory of Feed Biotechnology, State Key Laboratory of Animal Nutrition, College of Animal Science and Technology, China Agricultural University, Beijing, China; ^2^Prestage Department of Poultry Science, North Carolina State University, Raleigh, NC, United States

**Keywords:** anti-inflammatory activity, toll-like receptor, molecular dynamics simulation, lipopolysaccharide neutralization, intestinal barrier, NF-κB

## Abstract

Intestinal inflammatory disorders, such as inflammatory bowel disease (IBD), are associated with increased pro-inflammatory cytokine secretion in the intestines. Furthermore, intestinal inflammation increases the risk of enteric cancer, which is a common malignancy globally. Native anti-inflammatory peptides are a class of anti-inflammatory agents that could be used in the treatment of several intestinal inflammation conditions. However, potential cytotoxicity, and poor anti-inflammatory activity have prevented their development as anti-inflammatory agents. Therefore, in this study, we designed and developed a novel hybrid peptide for the treatment of intestinal inflammation. Eight hybrid peptides were designed by combining the active centers of antimicrobial peptides, including LL-37 (13-36), YW12D, innate defense regulator 1, and cathelicidin 2 (1-13) with thymopentin or the active center of thymosin alpha 1 (Tα1) (17-24). The hybrid peptide, LL-37-Tα1 (LTA), had improved anti-inflammatory activity with minimal cytotoxicity. LTA was screened by molecule docking and *in vitro* experiments. Likewise, its anti-inflammatory effects and mechanisms were also evaluated using a lipopolysaccharide (LPS)-induced intestinal inflammation murine model. The results showed that LTA prevented LPS-induced impairment in the jejunum epithelium tissues and infiltration of leukocytes, which are both histological markers of inflammation. Additionally, LTA decreased the levels of tumor necrosis factor-alpha, interferon-gamma, interleukin-6, and interleukin-1β. LTA increased the expression of zonula occludens-1 and occludin, and reduced permeability and apoptosis in the jejunum of LPS-treated mice. Additionally, its anti-inflammatory effect is associated with neutralizing LPS, binding to the Toll-like receptor 4-myeloid differentiation factor 2 (TLR4/MD-2) complex, and modulating the nuclear factor-kappa B signal transduction pathway. The findings of this study suggest that LTA may be an effective therapeutic agent in the treatment of intestinal inflammation.

## Introduction

Intestinal inflammation is a defensive response against infections and damage caused by microbiological toxins or noxious substances (1). Clinical symptoms of intestinal inflammation include abdominal pain, diarrhea, rectal bleeding, weight loss, malnutrition, and fever (2). Furthermore, intestinal patients, such as those with inflammatory bowel disease (IBD), have an increased risk of developing colorectal and small intestinal cancers (3–5). The mechanisms of intestinal inflammation as well as its progression to intestinal cancer have been extensively studied, focusing on dysregulation within the immune response and breakdown of the mucosal barrier (6).

Intestinal inflammation is treated with corticosteroids, specifically glucocorticoids (7, 8). This treatment can successfully decrease the production of pro-inflammatory cytokines and chemokines, cell adhesion molecules, and other key mediators of inflammation (9); however, prolonged use of corticosteroids is related to side effects, including impaired wound healing, mild hirsutism, linear growth inhibition, myopathy, osteoporosis, osteonecrosis, peptic ulcers, pancreatitis, and candidiasis (10, 11). Therefore, there is a need to identify and develop new drugs that have both the desired efficiency and improved safety.

In recent years, antimicrobial peptides (AMPs) have been reported to have anti-inflammatory effects (12–14). AMPs can not only directly interact with lipopolysaccharide (LPS) to inhibit the release of inflammatory cytokines (15, 16), but can also inhibit the translocation of nuclear factor-kappa B (NF-κB) to dampen the inflammatory response (13). As a result, AMPs are especially appealing candidates for the treatment of inflammatory disorders. LL-37 and YW12D effectively neutralize LPS; consequently, they have considerable potential for the treatment of LPS-induced inflammation (15, 17, 18). Cathelicidin 2 (CATH-2), a highly cationic (11^+^) chicken heterophil-derived peptide, inhibits IL-1β, and nitric oxide production in LPS-induced HD11 cells (19). Innate defense regulator (IDR)-1, one type of synthetic innate defense regulators, has protective activity against LPS-induced inflammation mediated by modulating NF-κB and mitogen-activated protein kinase (MAPK) signaling pathways (13, 20). Based on their previous anti-inflammatory activity observed, LL-37, IDR-1, CATH2, and YW12D were selected for further study.

Thymopentin (TP5) is a synthetic peptide consisting of five amino acid residues (21), and thymosin alpha 1 (Tα1) is a 28-amino acid peptide produced by thymic stromal cells (22, 23). Both TP5 and Tα1 exhibit similar immunoregulatory activity. They play an important role in regulating immunity, tolerance, and inflammation (24–26). TP5 and Tα1 exert their immune-modulating effect by interacting with Toll-like receptors (TLRs) and intracellular signaling pathways, such as NF-κB, MAPK, and myeloid differentiation primary response 88 (MyD88) pathways (27–29) Additionally, TP5 and Tα1 can counteract a pro-inflammatory cytokine storm and autoimmunity (24, 30, 31). Overall, TP5 and Tα1 exhibit immunoregulatory activity and low cytotoxicity (30, 32). Thus, they are commonly used in the clinic to treat various types of inflammatory diseases, such as cancer and infectious disease (21, 24, 33, 34).

The development of AMPs as potential anti-inflammatory drugs has faced several obstacles that are mainly attributed to their significant cytotoxicity toward eukaryotic cells, hampering their clinical development (19, 35, 36). In contrast, TP5 and Tα1 exhibit low cytotoxicity but relatively weak anti-inflammatory activity (30, 32). To improve the anti-inflammatory activity and reduce undesirable cytotoxic effects of native peptides, hybridization has been put forward. Hybridization is a simple and effective strategy for developing novel therapeutic peptides that combines the advantages of different native peptides (37). It has been reported that LL-37 (13-36) and CATH2 (1-13), which are the short peptides derived from native AMP, can effectively attenuate antigen- and pathogen-induced inflammation (19, 38, 39). Tα1 (17-24) also exhibited good immunoregulatory activity (40, 41). Thus, in the present study, we designed eight hybrid peptides by combining AMPs, including YW12D and IDR-1, or the active center of AMPs, including LL-37 (13-36) and CATH2 (1-13) with TP5 or the active center of Tα1 (17-24). The hybrid peptides were evaluated based on their anti-inflammatory activity and cytotoxicity. The best hybrid, based on these criteria, was screened by molecule docking and *in vitro* experiments. Likewise, its anti-inflammatory effect and mechanism were also evaluated using an LPS-induced murine model of intestinal inflammation.

## Materials and Methods

### Hybrid Peptide Design

The hybrid peptides were constructed by combining the active center of LL-37, YW12D, IDR-1, and CATH2 with the TP5 or the active center of Tα1.The amino acid sequences of the parental and hybrid peptides are listed in the Table 1.

**Table 1:** Key physicochemical parameters of parental and hybrid peptides.

**Peptides**	**Sequence**	**H^a^**
LL-37	LLGDFFRKSKEKIGKEFKRIVQRIKDFLRNLVPRTES	−0.559
YW12D	YVKLWRMIKFLR	0.167
IDR-1	KSRIVPAIPVSLL	1.046
CATH2	RWGRFLRKIRRFRPKVTITIQGSARF	−0.638
TP5	RKDVT	−1.608
Tα1	SDAAVDTSSEITTKDLKEKKEVVEEAEN	−1.029
LTP	IGKEFKRIVQRIKDFLRNLVPRTERKDVT	−0.8
LTA	IGKEFKRIVQRIKDFLRNLVPRTEKEKKEVVE	−0.894
YTP	YVKLWRMIKFLRRKDVT	−0.376
YTA	YVKLWRMIKFLRKEKKEVVE	−0.59
ITP	KSRIVPAIPVSLLRKDVT	0.289
ITA	KSRIVPAIPVSLLKEKKEVVE	−0.09
CTP	RWGRFLRKIRRFRRKDVT	−1.61
CTA	RWGRFLRKIRRFRKEKKEVVE	−1.635

a *The mean hydrophobicity (H) is the total hydrophobicity (sum of all residue hydrophobicity indices) divided by the number of residues*.

### Sequence Analysis of Hybrid Peptides

The mean hydrophobicity was calculated online using ProParam (ExPASy Proteomics Server: http://www.expasy.org/tools/protparam.html). The 3D structures of hybrid peptides LL-37-TP5 (LTP), LL-37-Tα1 (LTA), YW12D-TP5 (YTP), YW12D-Tα1 (YTA), IDR-1-TP5 (ITP), IDR-1-Tα1 (ITA), CATH2-TP5 (CTP), and CATH2-Tα1 (CTA) were built using I-TASSER (http://zhanglab.ccmb.med.umich.edu/I-TASSER/).

### Hybrid Peptides Scan by Molecule Docking

The constructed 3D structures of the hybrid peptides were then subjected to molecular docking. The initial structure for myeloid differentiation factor 2 (MD-2) was extracted from the crystal structure of the TLR4/MD-2 complex (PDB code: 2Z64). The initial MD-2/hybrid peptide complex was generated by ZDOCK3.0.2. For each hybrid peptide, a total of 3600 decoy structures were predicted through the rigid-binding option in ZDOCK; among these, the decoy with the lowest energy was chosen for the following flexible docking study. For each molecule, 100 docking runs were performed by flexpepdock (http://flexpepdock.furmanlab.cs.huji.ac.il/). The most plausible docking confirmation with the lowest score, which is calculated by the total Rosetta energy, was selected for scanning of the hybrid peptide.

### Peptides Synthesis

The hybrid peptides, LTP, LTA, CTP, and YTP, which were selected via molecule docking, and their parental peptides, LL-37, CATH2, YW12D, TP5, and Tα1, were synthesized and purified by KangLong Biochemistry (Jiangsu, China). The purity of all peptides was higher than 95%, as determined by high performance liquid chromatography (HPLC) and mass spectrometry. The peptides were dissolved in endotoxin-free water and stored at −80°C.

### Cell Culture

Mouse macrophage cell line (RAW264.7) was purchased from the Shanghai Cell Bank, the Institute of Cell Biology, China Academy of Sciences (Shanghai, China). The cells were cultured in Dulbecco's modified Eagle's medium (DMEM; Hyclone) supplemented with 10% (v/v) fetal bovine serum (FBS; Bioscience) and 1% (v/v) penicillin/streptomycin (Hyclone) at 37°C in a moist atmosphere (5% CO_2_, 95% air).

### Cell Viability Assay

The viability of peptide-treated RAW264.7 cells was determined using the Cell Counting Kit-8 (CCK-8) Assay Kit (Dojindo) (42). RAW264.7 cells were pre-seeded on a 96-well plate at a density of 3 × 10^4^ cells/mL in 100 μL DMEM medium overnight. The cells were either treated with various concentrations of peptides or without peptides at 37°C and 5% CO_2_ for 24 h. Each well was incubated with 10 μL CCK-8 solution for 4 h in the darkness. Then, the absorbance at 450 nm was measured using a microplate reader. Cell viability was calculated as:

$$\begin{array}{l} Cell\;viability\;\left(\%\right)=\left(OD450\;\left(sample\right)/OD450\left(control\right)\right)\times 100\% \end{array}$$

where OD_450(sample)_ is the absorbance at 450 nm of the cells with peptides treated and OD_450(control)_ is the absorbance at 450 nm of the cells without peptides treated.

### Animal Model

Seventy-two C57/BL6 male mice (6–8 weeks of age) were purchased from Charles River (Beijing, China). Mice were maintained in a specific pathogen free (SPF) environment at 22 ± 1°C with relative 55 ± 10% humidity, and the assays were performed in conformity with the laws and regulations for live animal treatment at China Agricultural University.

The mice were randomly distributed into six groups (*n* = 12 each): control, LPS (*E. coli*, O111:B4, Sigma-Aldrich, USA) treatment, LL-37 pretreatment followed by LPS treatment (LL-37 + LPS), Tα1 pretreatment followed by LPS treatment (Tα1 + LPS), LTA pretreatment followed by LPS treatment (LTA + LPS). Different peptides (10 mg/kg mouse weight) were injected intraperitoneally once daily for 7 days, whereas an equal volume of sterile saline was injected intraperitoneally to the control and LPS-treated groups. On day 7, mice in LPS, LL-37 + LPS, Tα1 + LPS, and LTA + LPS groups were intraperitoneally injected with LPS (10 mg/kg mouse weight) 1h after saline or the peptides treatment, and the control group was intraperitoneally injected with an equal volume of saline. The mice were then euthanized by cervical dislocation 6 h after intraperitoneal injection of LPS or saline, and samples of the intestines were collected for analysis. The body weights of the mice were recorded before and after the experiment.

### Histopathology and Immunohistochemistry

Intestinal tissues samples from the jejunum were fixed in 4% paraformaldehyde immediately after the mice were euthanized. After embedding, the tissues were sectioned (5 μm) using an RM2235 microtome (Leica) and stained with hematoxylin-eosin (H&E). Images were acquired using a DM3000 microscope. LPS-induced intestinal injury was evaluated using Chiu's score (43) according to changes of the villus and glands of the jejunal mucosa. Villus height and crypt depth were measured using CaseViewer software.

For immunohistochemical analysis of CD177^+^, nonspecific binding sites were blocked with PBS containing 1% w/v BSA for 1 h. Anti-CD177^+^ antibodies (Santa, USA) were added at a dilution of 1:100 and incubated overnight at 4°C. Samples were washed five times in PBS and treated with horse-radish peroxidase (HRP)-conjugated rabbit anti-goat IgG (JIR, USA) at ratio of 1:100; samples were left to incubate at 4°C for 1 h. After washing with PBS, 3,3′-diaminobenzidine (DAB; DAKO, USA) was added, and the slides were counterstained with Harris hematoxylin. Finally, the samples were dehydrated in an alcohol gradient (70–100%), and cleared in xylene. All slides were mounted in neutral balsam.

The apoptotic cells in the jejunal sections were detected via a commercial the terminal deoxynucleotidyl transferase mediated dUTP nick end labeling (TUNEL) staining kit according to the manufacturer's instruction (Roche, Indianapolis, IN, USA). The sections were co-stained with the DAPI (Servicebio, Wuhan, China). The number of apoptotic cells was counted in four to six randomly selected fields at 200 × magnification.

### Transmission Electron Microscopy (TEM)

The tight junctions (TJs) between gut epithelial cells were observed by TEM. A jejunum specimen was excised with a scalpel and fixed in 2.5% glutaraldehyde for 4 h at 4°C. Afterwards, the specimens were treated with osmic acid and embedded in epon. Ultrathin sections were acquired using a diamond knife, and stained with uranyl acetate and lead citrate before visualization by TEM (Model H-7650, HITACHI, Japan).

### Measurement of Transepithelial Electrical Resistance (TEER)

The TEER values of intestinal membranes were assessed by an *in vitro* diffusion chamber method using stripped mouse jejunal membranes. The underlying muscularis of the jejunal membranes were removed, and the jejunal segments were mounted in a diffusion chamber with an exposed surface area of 1.78 cm^2^. Ussing chambers were equipped with two pairs of electrodes connected to the chambers by 3 M KCl/3.5% agar bridges. Each side of the chamber was bubbled with a mixture of 5% CO_2_ and 95% O_2_ to maintain the viability of the jejunal membranes. The temperature was maintained at 37°C during the experiment by a circulating water bath. The potential difference (PD) and the short-circuit current (Isc) were measured, and then, total electrical resistance (RT) was calculated by Ohm's law, that is RT = PD/Isc (44).

### ELISA

RAW264.7 cells were treated with or without 10 μg/mL peptides for 1h before the addition of 100 ng/mL LPS and further incubation for 12 h at 37°C. Levels of tumor necrosis factor-alpha (TNF-α), interleukin-6 (IL-6), and IL-1β were detected in culture supernatant and jejunum, respectively. In addition, the level of interferon-gamma (IFN-γ) was detected in the jejunum. ELISA was performed using a commercial ELISA kit (eBioscience, San Diego, USA).

The activities of myeloperoxidase (MPO) in the jejunum were detected using ELISA kits (Boster Wuhan, China) according to the manufacturer's instructions.

### Western Blotting

Whole protein of intestinal tissues was obtained with the whole protein extraction kit (KeyGEN Biotech, Nanjing, China) according to the manufacturer's instructions. The protein concentration was measured via the BCA kit (KeyGEN Biotech, Nanjing, China). Protein samples (40 μg protein/lane) were separated by 10% sodium dodecyl sulfate-polyacrylamide gel electrophoresis (SDS-PAGE) and transferred to polyvinylidene difluoride (PVDF) membranes (Bio-Rad). The membranes were blocked with 5% nonfat dried milk in 0.05% TBST and immunoblotted with primary specific antibodies against inhibitor of κB (IκB)-α, p-IκB-α, IκB kinase (IKK)-β, p-IKK-β, NF-κB (p65), p-NF-κB (p-p65), zonula occluden-1 (ZO-1), occludin, and β-actin (Santa Cruz, CA, USA). After washing with TBST, membranes were incubated with anti-mouse/rabbit HRP-conjugated secondary antibodies (HuaAn, Hangzhou China). The proteins were detected with SuperSignal West Femto maximum sensitivity substrate (Pierce Biotechnology) and visualized using a ChemiDoc MP Imaging System (Bio-Rad, Hercules, CA, USA).

### Neutralization of LPS

The neutralization of LPS by the peptides was assessed using a quantitative Chromogenic End-point Tachypleus Amebocyte Lysate (CE TAL) assay via the QCL-1000 kit (Xiamen, China). A constant concentration of LPS (1.0 EU/mL final concentration; *E. coli*, O111:B4, Sigma-Aldrich, USA) was incubated with various concentrations of the peptides or polymyxin B (PMB) (0–64 μg/mL final concentration; Sigma-Aldrich, USA) at 37°C for 15 min in the wells of pyrogenic sterile microliters plates. The 100 μL aliquots concentrate of limulus amebocyte lysate reagent was added and incubated at 37°C for 6 min. On the addition of chromogenic substrate, yellow color appeared. The reactions were stopped by adding 25% HCl, and then the absorbances measured at 540 nm (45).

The level of LPS in the plasma were detected using QCL-1000 kit (Xiamen, China) according to the manufacturer's instructions.

### Molecular Dynamics Simulation

The crystallographic structure of the TLR4/MD-2 complex was taken from PDB bank (PDB code: 2Z64), and the initial structure for MD-2 was extracted from the crystal structure of the TLR4/MD-2 complex. The missing hydrogen atoms were added under pH 7.0 by Maestro (46). The docking pose was determined by RosettaDock (version 3.5), and the pose with the lowest score (total Rosetta energy for this model) was selected for further analysis.

The best binding pose of LTA with MD-2 was refined using a molecular dynamics (MD) simulation with AMBER14 (47, 48). The force fields used for the simulation were GAFF and FF14SB, and the system was solvated with TIP3P water molecules in a cubic box with a minimum distance of 10 Å between the protein and the edge of the box. Na^+^ and Cl^−^ atoms were added to mimic the physiological conditions and neutralize the system. The system was first minimized with 5000 steps by the conjugate gradient algorithm, following by heating gradually over 100 ps. Subsequently, the volume of the system was adjusted under a constant number, pressure, and temperature (NPT) ensemble. Afterwards, a 60 ns MD simulation was performed under constant number, volume, and temperature (NVT) ensemble.

Based on the 300 snapshots extracted from the last 40 ns of the equilibrated MD simulation, the long-range electrostatic interactions were calculated by the Particle-mesh Ewald (PME) method (49), and the binding energy was calculated by the molecular mechanics Poisson-Boltzmann accessible surface area (MM-PBSA) method (50).

### Flow Cytometry

RAW264.7 cells were treated with PBS, anti-mouse mAbTLR4/MD-2 complex (MTS510 Ab) (eBioscience, San Diego, USA) or isotype control (IgG) (eBioscience, San Diego, USA) for 1 h at 4°C before staining with 10 μg/mL N-terminus fluorescein isothiocyanate (FITC)-labeled LTA. The cells were then harvested by trypsin and washed five times with PBS. The average FITC intensity of the cells was measured via flow cytometry.

### Analysis of Confocal Laser-Scanning Microscopy

To verify TLR4/MD-2 as the pattern recognition receptor, RAW264.7 cells were treated with PBS, MTS510 Ab or isotype control (IgG) (eBioscience, San Diego, USA) for 1 h at 4°C. Afterwards, RAW264.7 cells were incubated with N-terminus FITC-LTA at 10 μg/mL for 1 h at 4°C in the dark. Then, the cells were washed with PBS, fixed with paraformaldehyde, and rinsed twice with PBS. The cell nuclei were stained with DAPI (diluted 1:500 in PBS) (Sigma, USA) for 5 min, and the cells were rinsed six times with PBS. The above cells were spread on a glass slide, fixed, and observed via a Leica TCA sp5 confocal microscope (Germany).

### Statistics

All data are expressed as mean values ± standard deviation from at least 3 independent experiments. Statistical comparisons were carried out by Student's t test and ANOVA test with GraphPad Prism v6 software (La Jolla, California). Significance was claimed with *P* values ≤ 0.05. NS: *P* > 0.05, ^*^P ≤ 0.05, ^**^P ≤ 0.01, ^***^P ≤ 0.001, and ^****^P ≤ 0.0001.

## Results

### Selection of Anti-inflammatory Peptides by Molecular Docking

**Figure 1:**
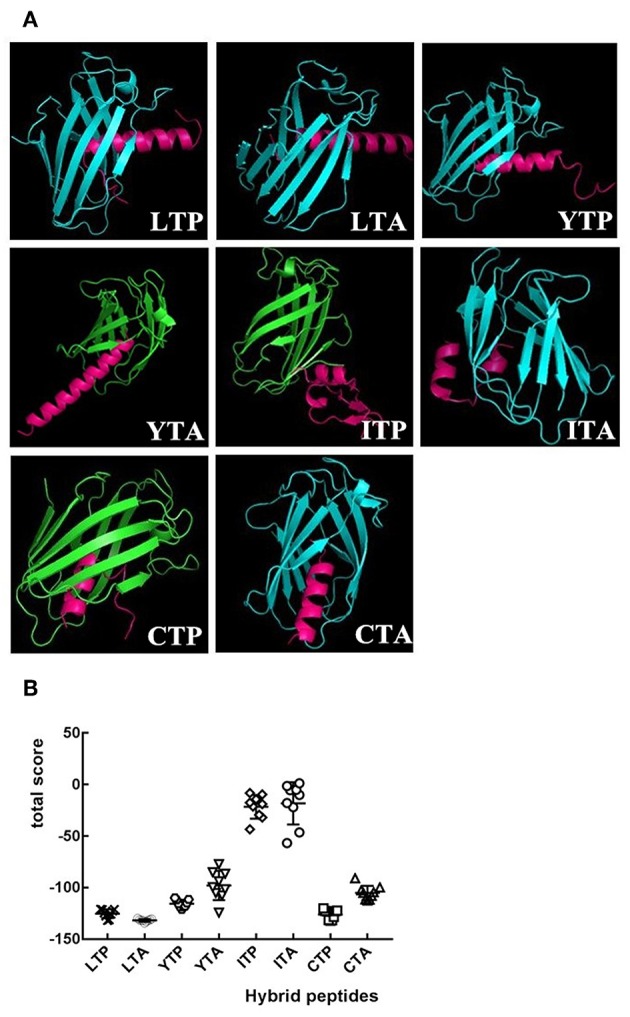
The overall structure of hybrid peptide-MD-2 Complex. **(A)** Shows the views of the hybrid peptides (red) binding to MD-2 (blue or green). **(B)** An energy plot with 10 out of 100 decoy structures from an MD-2 docking study by Flexpepdock. Data are given as mean values ± standard.

As an initial screen of the anti-inflammatory peptides, the binding modes of the eight hybrid peptides to MD-2 were analyzed by molecular docking. As shown in Figures 1A,B, of the eight hybrid peptides, LTP, LTA, YTP, and CTP had more favorable docking scores to MD-2, and the total social of them was lower than −100.

### Cytotoxicity on RAW264.7 Macrophage Cells

**Figure 2:**
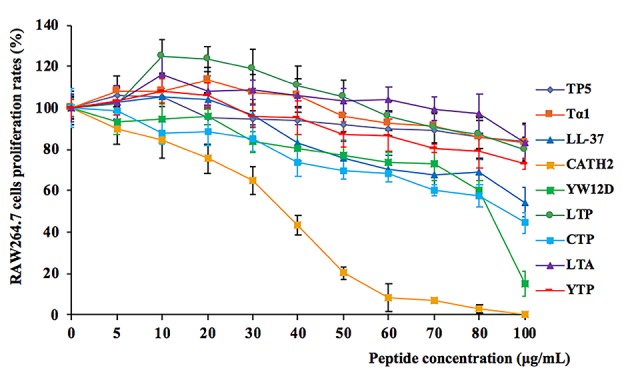
Cell proliferation rates of RAW264.7 cells in the absence or presence of parental peptides and hybrid peptides. RAW264.7 cells were pre-seeded in DMEM medium overnight. The cells were treated with various concentrations of peptides or without peptides at 37°C and 5% CO_2_ for 24 h. They were incubated with CCK-8 solution for 4 h, and then, they were measured at 450 nm. Data are given as mean values ± standard deviation from 8 biological replicates.

The cytotoxic activity of the peptides on RAW264.7 macrophage cells was evaluated by CCK-8 assay, and the results are shown in Figure 2. Among the initial selected hybrid peptides and their parental peptides, LTP, and LTA exhibited the lowest cytotoxic activity, and the cell viability of the lower doses LTP- and LTA-treated cells was greater than 100%. Meanwhile, all the selected hybrid peptides showed lower cytotoxicity than their parental peptides. The cell viability of the peptide-treated RAW264.7 cells was greater than 80%. These data indicated that at a concentration of 10 μg/mL, all of the peptides were minimally cytotoxic to RAW264.7 cells and suitable for the following experiments.

### Inhibition of Cytokine Release From LPS-Stimulated RAW264.7 Cells

**Figure 3:**
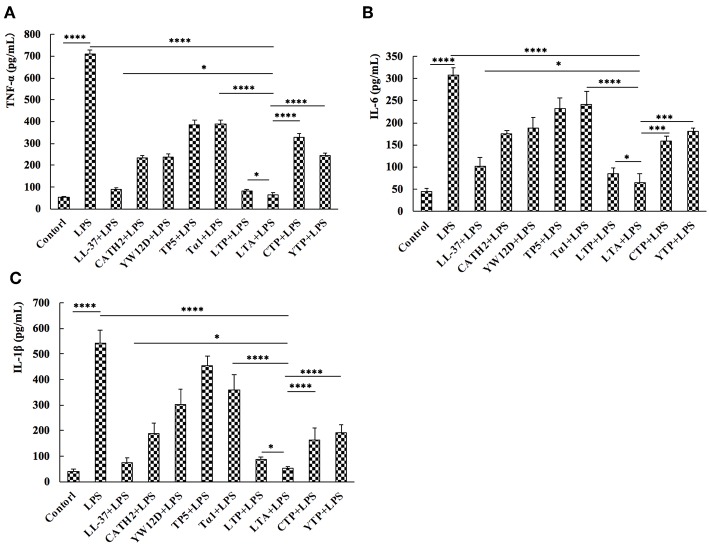
Hybrid peptides suppress the LPS-induced cytokine secretion. 12 h after treatment with 100 ng/mL LPS in the presence or absence of peptides, protein levels of TNF-α **(A)**, IL-6 **(B)**, and IL-1β **(C)** in RAW264.7 cells were quantified by ELISA. Data are given as mean values ± standard deviation from 3 biological replicates. NS: *P* > 0.05, _*_P ≤ 0.05, ^***^P ≤ 0.001, and ^****^P ≤ 0.0001.

To evaluate the anti-inflammatory effect of the hybrid peptides, the mouse macrophage cell line, RAW264.7, was used as a model. ELISA results show that all parental peptides and hybrid peptides were potent inhibitors of pro-inflammatory cytokine release (Figure 3). LPS caused a significant elevation in TNF-α, IL-6, and IL-1β levels compared to untreated RAW264.7 cells. Compared to the other tested peptides, 10 μg/mL of LTA in the presence of LPS caused a remarkable decrease in the release of TNF-α (Figure 3A), IL-6 (Figure 3B), and IL-1β (Figure 3C). Collectively, these data suggest that LTA is suitable for further anti-inflammatory experiments.

### Effect of Hybrid Peptides on Body Weight

**Figure 4:**
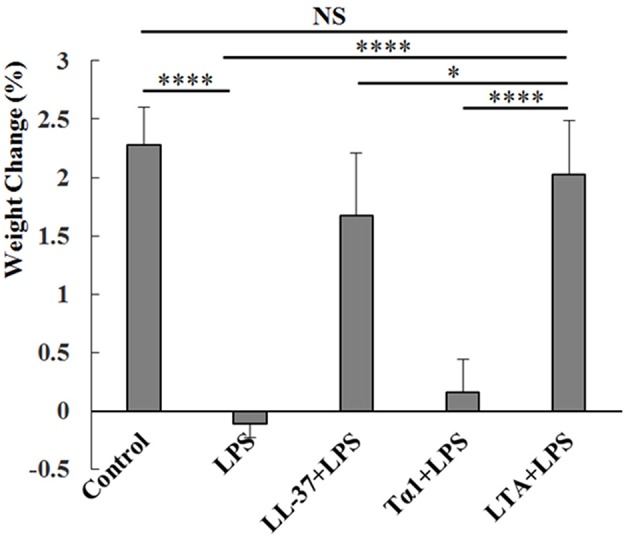
Protective effects of LTA on body weight. Different peptides (10 mg/kg) were injected into the mice once daily for 6 days, whereas the control and LPS-treated groups were injected with an equal volume of sterile saline. On day 6, mice in the LPS and peptide-pretreated groups were injected with LPS (10 mg/kg) 1 h after the peptide or saline treatment. The control group was injected with an equal volume of saline. The body weights of the mice were recorded before and after the experiment. Data are given as mean values ± standard deviation from 12 biological replicates. NS: *P* > 0.05, ^*^P ≤ 0.05, and ^****^P ≤ 0.0001.

As expected, LPS treatment resulted in weight loss. Mice in the LPS-treated group showed significant weight loss compared to the control group. Mice in the LTA-pretreated group recovered their weight loss rapidly (Figure 4). Based on the weight-loss recovery, the hybrid peptide, LTA, appears to be more potent than the parental peptides (Figure 4).

### The Protective Effects of LTA Against LPS-Induced Damage in Jejunum Tissue

**Figure 5:**
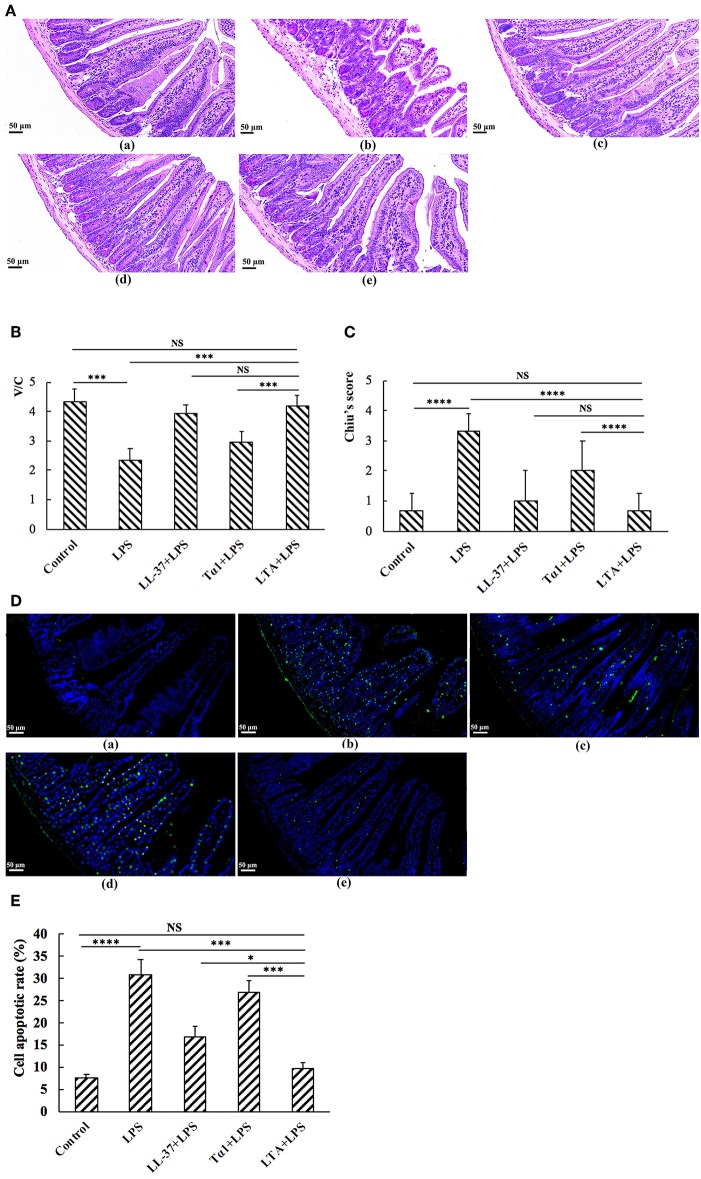
The protective effects of LTA against LPS-induced clinical symptoms in mouse jejunum. Representative H&E-stained sections from (**A**,a) control, (**A**,b) LPS, (**A**,c) LL-37 + LPS, (**A**,d) Tα1 + LPS, (**A**,e) LTA + LPS. Original magnification 200 ×. **(B)** The effect of LTA on Chiu's scores. Chiu's score is comprised of changes of the villus and glands of the jejunal mucosa **(C)** The effect of LTA on the ratio of villus height to crypt depth (V/C) of the jejunum. **(D)** TUNEL staining of jejunal tissues. Original magnification 200 ×. (**D**,a) Control, (**D**,b) LPS, (**D**,c) LL-37 + LPS, (**D**,d) Tα1 + LPS, (**D**,e) LTA + LPS. **(E)** The number of apoptotic cells in 4–6 randomly selected fields was counted according to the number of positive green cells and the average calculated. Data are given as mean values ± standard deviation from 12 biological replicates. NS: *P* > 0.05, ^*^P ≤ 0.05, ^***^P ≤ 0.001, and ^****^P ≤ 0.0001.

Mice in the LPS group had significantly more macroscopic inflammation than those in the control and LTA-pretreated groups. Compared to the control, histological examination of the jejunum tissue in the LPS group showed considerable tissue injury (Figure 5A) and a decreased villus height to crypt depth (V/C) ratio (Figure 5C). Overall, the LPS-induced intestinal damage was significantly attenuated by LTA pretreatment; Chiu's score was restored from 3.33 ± 0.58 to 0.67 ± 0.58, and the V/C value was restored from 2.35 ± 0.389 to 4.19 ± 0.364 (*p* ≤ 0.05) (Figure 5B). Based on the Chiu's score, the newly designed hybrid peptide appears to be more potent than the parental peptides (Figure 5B). In addition, the V/C value in the LTA-pretreated group was markedly increased compared to that in the Tα1-pretreated group. No statistical significance was found among the LTA-pretreated group compared to the LL-37-pretreated group (Figure 5C).

As shown by TUNEL staining, apoptosis of the LPS-treated group was significantly higher than that of the control group, as quantified by the apoptosis index (Figures 5D,E). Compared to the LPS-treated group, pretreatment with LTA in LPS-administered mice significantly reduced the apoptosis index (Figures 5D,E). In addition, the apoptosis index in the LTA-pretreated group was markedly decreased compared to the parental peptides.

**Figure 6:**
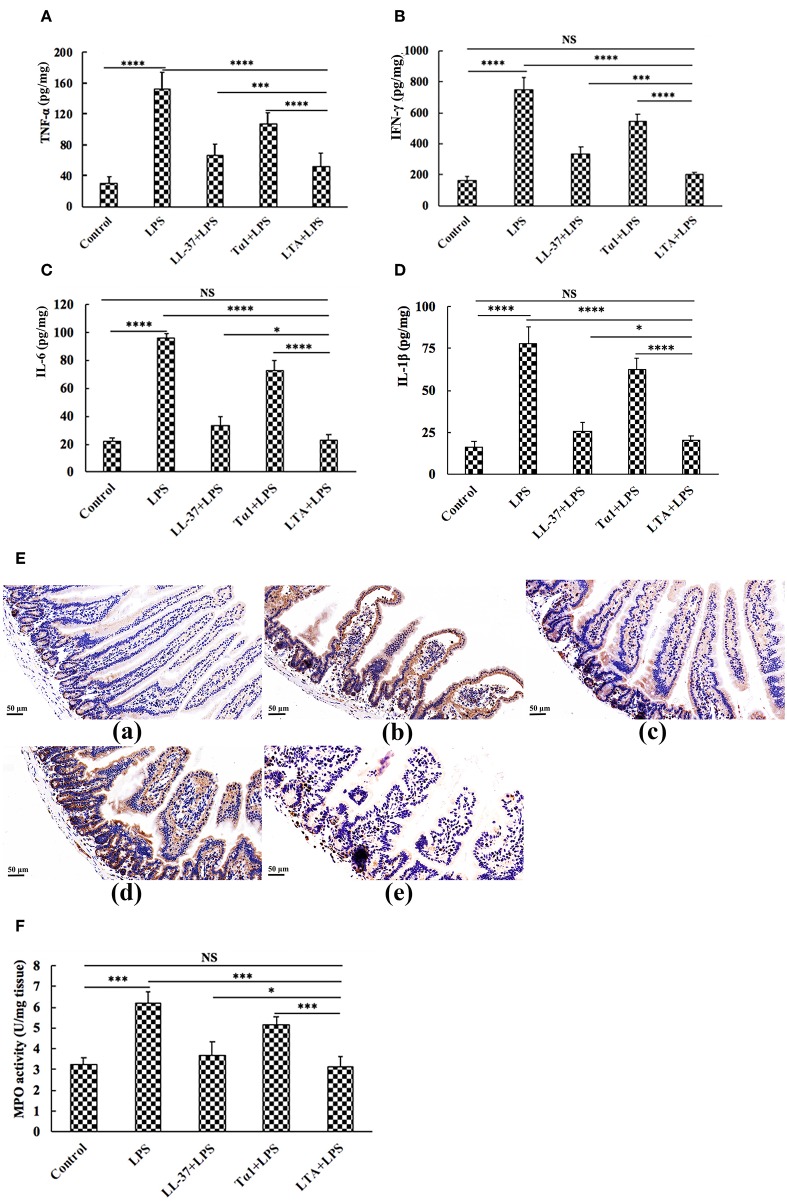
The protective effects of LTA on the inflammatory response. ELISA for TNF-α **(A)**, IFN-γ **(B)**, IL-6 **(C)**, and IL-1β **(D)** in jejunal tissues. **(E)** Representative images of CD177^+^. Original magnification 400 ×. Formalin-fixed, paraffin-embedded 5-mm cross-sections were stained with a primary Ab to CD177^+^. (**E**,a) control, (**E**,b) LPS, (**E**,c) LL-37+LPS, (**E**,d) Tα1+LPS, (**E**,e) LTA+LPS. Enzymatic activities of MPO were measured **(F)**. Data are given as mean values ± standard deviation from 12 biological replicates. NS: *P* > 0.05, ^*^P ≤ 0.05, ^***^P ≤ 0.001, and ^****^P ≤ 0.0001.

To characterize the protective effect of LTA against inflammation in LPS-induced mice, the secretion of the inflammatory markers, TNF-α, IFN-γ, IL-6, and IL-1β were evaluated by ELISA. Administration of LPS caused a significant elevation of these pro-inflammatory cytokines in the jejunum compared to the control group (Figures 6A–D). Figures 6A–D shows that all peptides attenuated TNF-α, IFN-γ, IL-6, and IL-1β secretion. Meanwhile, mice in the LTA-pretreated group had significantly lower TNF-α, IFN-γ, IL-6, and IL-1β concentrations than those in the Tα1- or LL-37-pretreated groups.

The infiltration of CD177^+^ cells into jejunal tissue was detected via immunohistochemistry. In contrast to minimal infiltration of neutrophils into the jejunum of control mice, LPS triggered increased infiltration of CD177^+^ neutrophils into the jejunal lesion area (Figure 6E). Pretreatment with the peptides reduced the infiltration of neutrophils compared to the group treated with LPS alone (Figure 6E). LTA, the most active peptide, reduced this effect to the basal level.

MPO (an indicator of jejunal infiltration by leukocytes) activity in the jejunum tissue from LPS treated mice was significantly increased compared to control mice. LTA pretreatment showed markedly decreased MPO activity compared to the LPS-treated group (Figure 6F). Moreover, MPO activity in the LTA-pretreated group was markedly decreased compared to Tα1 and LL-37 pretreatment (Figure 6F). Collectively, all of these results support the assertion that LTA is the most active peptide against LPS-induced impairment of mice, and it is suitable for further experiments.

### LTA Prevented the LPS-Stimulated Disruption of Intestinal TJ Structure and Function

**Figure 7:**
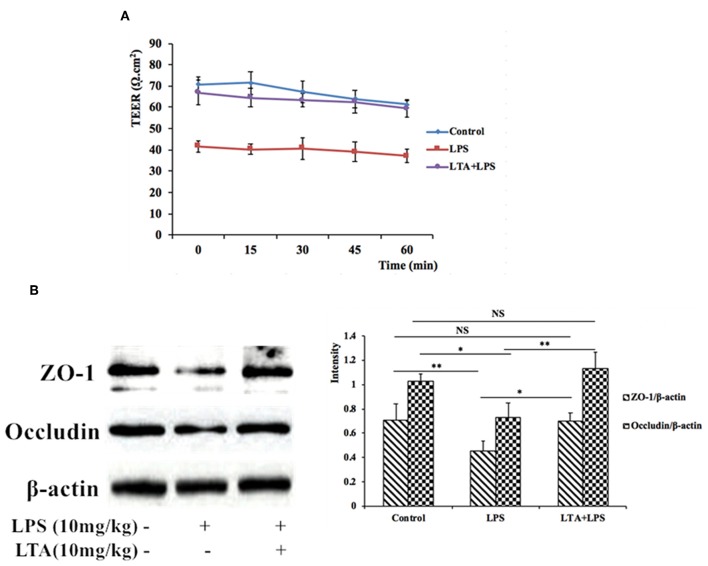
The protective effects of LTA on the intestinal barrier. **(A)** TEER of mouse jejunal epithelium was measured *ex vivo* in Ussing chambers. **(B)** Expression of TJ proteins was determined by western-blot. Data are given as mean values ± standard deviation from at least 3 biological replicates. NS: *P* > 0.05, ^*^P ≤ 0.05, and ^**^P ≤ 0.01.

To assess the functional integrity of mouse intestinal epithelium under *ex vivo* conditions, TEER measurements were performed for 60 min. Compared to the control group, the TEER values in the LPS-treated group declined remarkably (Figure 7A), indicating an increase in permeability. However, pretreatment with LTA resulted in a significant protective effect; the TEER values in the LTA-pretreated group were similar to those in the control group (Figure 7A). These results suggest that LTA minimizes LPS-induced intestinal epithelial hyper permeability.

**Figure 8:**
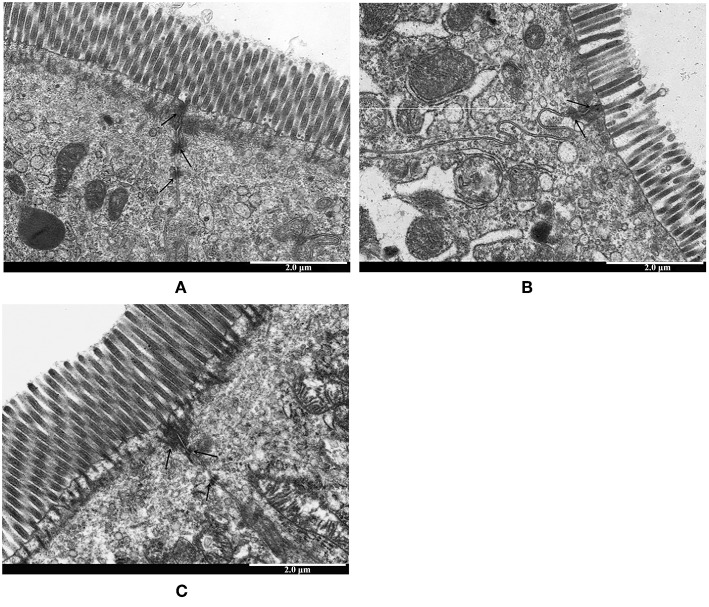
The protective effects of LTA on intestinal TJs structure. TJs structure in the jejunal epithelium was confirmed by transmission electron microscope (TEM). Original magnification 20000 × **(A)** control, **(B)** LPS, **(C)** LTA+LPS. The wider intervals (black arrowheads) between the intestinal epithelial cells are indicated.

To investigate the protective effects of LTA on the LPS-stimulated disruption of TJs, TJ marker (ZO-1 and Occludin) levels were determined by western blotting. Compared to control group, the expression of ZO-1 and Occludin was down-regulated in mice treated with LPS alone (Figure 7B). However, the expression of these TJ markers in the LTA-pretreated group was significantly higher than that in the LPS-group (Figure 7B). These data suggest that LTA maintains the integrity of the junction complex. In addition, TEM, which was used to determine the TJs between gut epithelial cells, supported the protective effect of LTA against LPS-induced damage in jejunum tissues (Figure 8).

### LTA Effects on the NF-κB Signaling Pathway in LPS-Induced Mice

**Figure 9:**
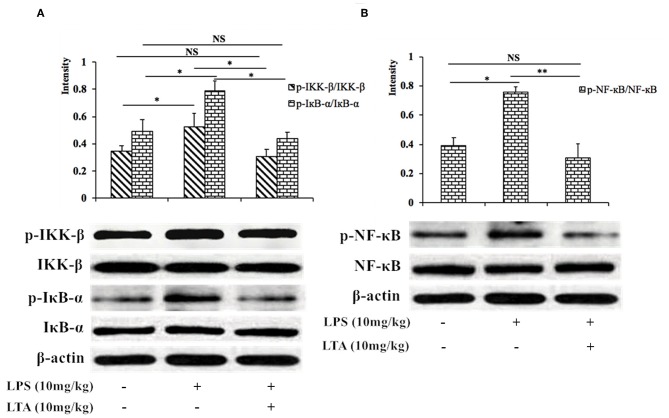
Inhibitory effect of LTA on the NF-κB signaling pathways in mice. Phosphorylated and total protein levels of IKK-β and IκB-α **(A)**, NF-κB **(B)**, and β-actin from jejunal tissues were measured by western blot analyses. Data are given as mean values ± standard deviation from 3 biological replicates. NS: *P* > 0.05, ^*^P ≤ 0.05, and ^**^P ≤ 0.01.

Next, we investigated the NF-κB signaling pathway in LPS-induced mice pretreated with or without LTA to determine the mechanism by which LTA induces its anti-inflammatory activity. The phosphorylation of IKK-β, IκB-α, and NF-κB increased significantly after stimulation with LPS, but the phosphorylation decreased in the group pretreated with LTA (Figure 9). These results suggest that one mechanism by which LTA modulates the immune system in mice is via the NF-κB pathway.

### LPS Neutralization Activity of LTA *in vitro* and *in vivo*

**Figure 10:**
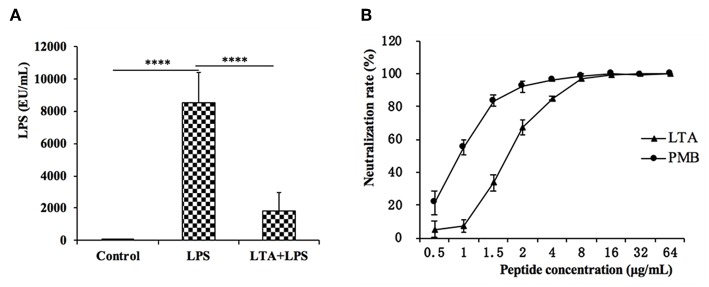
LPS neutralization activity of LTA *in vitro and in vivo*. **(A)** The LPS concentration in the mice plasma. **(B)** *In vitro* LPS neutralization by LTA. Binding of LTA (shown as triangles) or PMB (shown as a circle) binding to LPS was determined using the chromogenic *in vitro* TAL assay. Data are given as mean values ± standard deviation from 3 biological replicates. NS: *P* > 0.05, ^****^P ≤ 0.0001.

The plasma LPS concentration in the mice was evaluated by the CE TAL test. In the LPS-treated group, the plasma LPS level sharply increased, but LTA significantly reduced the plasma LPS level (Figure 10A).

To test whether LTA neutralized LPS, an LPS neutralization activity was performed *in vitro*. As shown in Figure 10B, LTA inhibited the activation of tachypleus amebocyte lysate in a dose-dependent manner. The LPS neutralization activity LTA is similar to PMB, a cyclic hydrophobic peptide known to bind LPS (51), and LTA could completely neutralize LPS at 8 ug/mL or more.

### The Specific Binding of LTA to TLR4/MD-2

**Figure 11:**
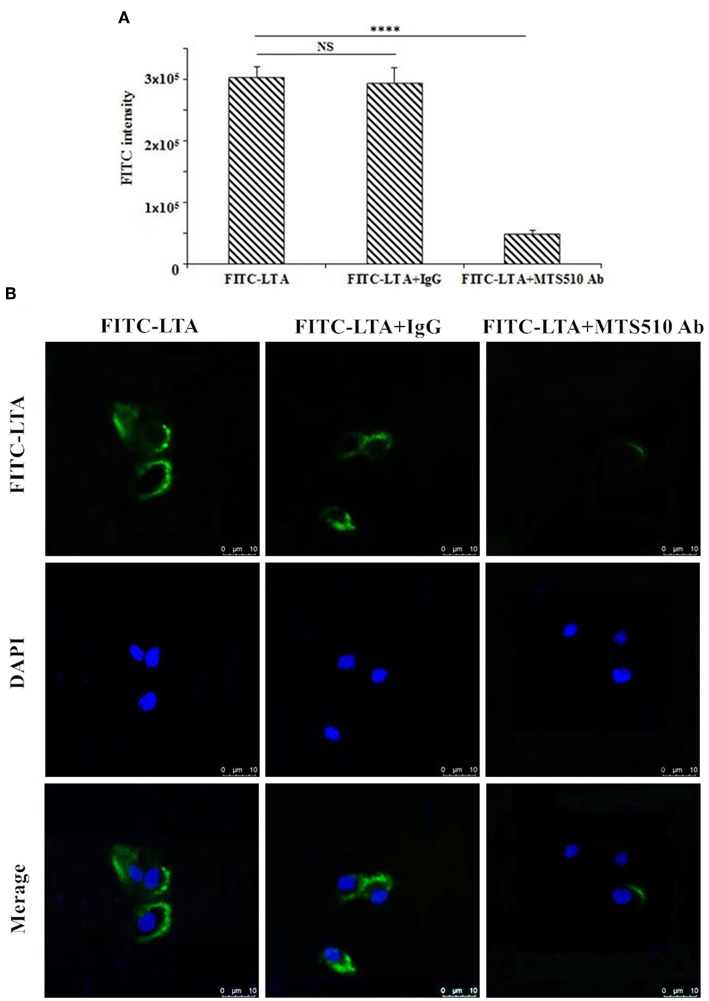
TLR4/MD-2-dependent staining of RAW264.7 cells with FITC-LTA. **(A)** RAW264.7 cells were treated with PBS, anti-mouse mAbTLR4/MD-2 complex (MTS510 Ab) or isotype control (IgG) for 1 h at 4°C before staining with 10 μg/mL. The cells were then harvested by trypsin and washed five times with PBS. The above cells were analyzed by the flow cytometry. **(B)** Cells were treated with PBS, MTS510 Ab, or IgG before FITC-LTA staining for confocal laser scanning microscopy analysis. Bar, 10 μm. Data are given as mean values ± standard deviation from 3 biological replicates. NS: *P* > 0.05, ^****^P ≤ 0.0001.

**Figure 12:**
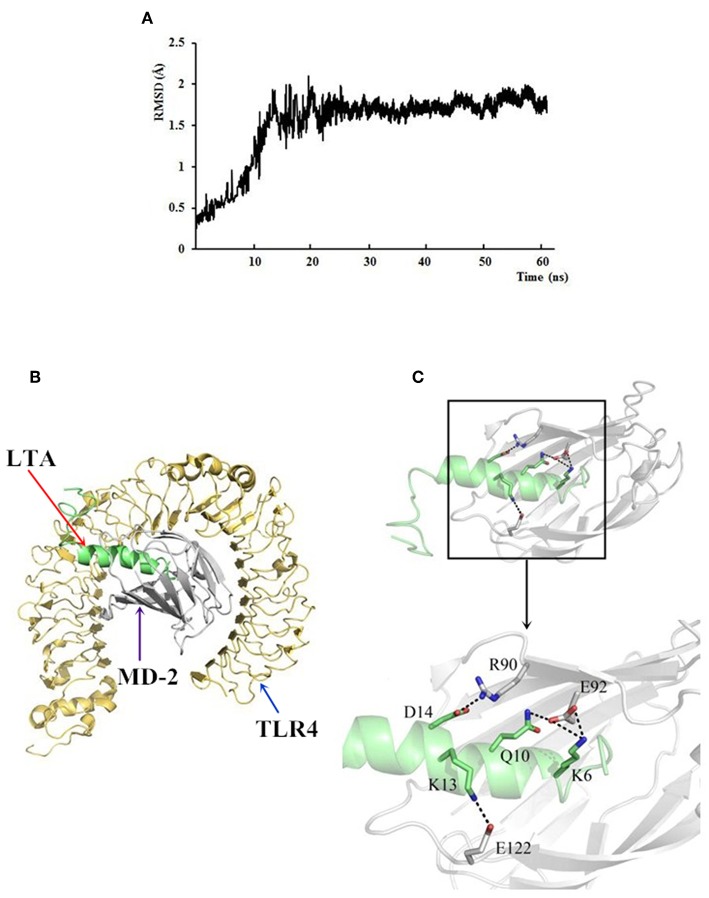
The docking results of LTA to the TLR4/MD-2 complex. **(A)** Time evolution of RMSD during molecular dynamics simulation. **(B)** LTA is bound to the hydrophobic pocket of MD2. The yellow ribbons represented TLR4, gray ribbons represented MD2, and green ribbons represented LTA. **(C)** A close-up view of LTA binding to the MD-2. Residues involved in the interaction between LTA and MD-2 are displayed.

To determine the recognition receptor, a binding assay of LTA to a receptor on the plasma membrane was performed via flow cytometry (Figure 11A) and confocal laser-scanning microscopy (Figure 12B). FITC-LTA caused a significant increase in the fluorescence intensity of RAW264.7 cells compared to the intensity of cells blocked with an anti-mouse TLR4/MD-2 mAb. Meanwhile, this increased fluorescence intensity was not attenuated by IgG (Figure 12A). Similar results were also seen via confocal laser-scanning microscopy (Figure 11B).

To further predict binding of LTA to the TLR4/MD-2 complex, MD simulations were performed. Based on the root-mean square deviation (RMSD) values of TLR4/MD-2 (Figure 12A), the MD simulation was fully equilibrated during the full 60 ns. A total of 300 snapshots was taken from the last stable 40 ns of the MD simulation. The calculated binding free energy, which was well correlated with the determined binding affinities, is shown in Table 2. The binding energy of LTA to MD-2 calculated by MM-PBSA method was −996.2 kJ/mol. The interface of MD-2 that is bound to LPS was compared to that of LTA. There was a common hydrophobic pocket on MD-2 where both LPS (52) and LTA interacted with the protein (Figures 12B,C). The interaction between LTA and MD-2 was principally mediated by salt-bridges and hydrogen bonds between Asp14 (LTA) - Arg 90 (MD-2), Lys 6 and Gln 10 (LTA) - Glu 92 (MD-2), and Lys 13 (LTA) – Glu 122 (MD-2) (Table 3). This is consistent with the flow cytometry and confocal laser-scanning microscopy results that suggest that LTA blocks LPS binding to TLR4/MD2 complex, resulting in the LPS-antagonizing effects.

**Table 2:** Key parameters of interactions between LTA and MD-2.

**Interaction pair**	**Number of salt-bridges**	**Number of hydrogen bonds**	**Interaction surface (Å^**2**^)**	**Binding free energy (kj/mol)**
MD2…LTA	4	5	343	−996.2

**Table 3:** Distances and salt-bridges between the binding residues of LTA and MD-2.

**Interaction pair MD2…LTA**	**Distance (Å)**	**Salt-bridges**
R_90_-NH_2_…D_14_-OD_1_	3.30	**+**
E_122_-OE_2_…K_13_-NZ	3.00	**+**
E_122_-OE_1_…K_13_-NZ	2.94	**+**
E_92_-OE_1_…Q_10_-NE_2_	3.00	**–**
E_92_-OE_1_…K_6_-NZ	3.03	**+**
E_92_-OE_2_…K_6_-NZ	3.19	**+**

*The distance and salt-bridges of interaction pairs are extrapolated from the LTA/MD-2 molecular dynamics simulation. “+” means existence, and “–” means not determined*.

## Discussion

LPS, a major component of the cell wall of Gram-negative bacteria, is released by antibiotic intake and can lead to intestinal inflammation. Intestinal inflammation is a chronic inflammatory disease associated with engagement of the immune response by increased pro-inflammatory cytokine secretion in the intestines (53, 54). Historically, intestinal inflammation is more common in western countries than Asia; the increasing incidence in Asia is likely due to the influence of a high-fat diet (55). Notably, intestinal inflammation increases the risk of enteric cancer, which is a commonly malignancy globally (56).

Native anti-inflammatory peptides are a class of anti-inflammatory agents that may be useful in the treatment of a range of inflammatory diseases (13, 18, 25). However, several concerns, such as potential cytotoxicity (35), poor anti-inflammatory activity based on peptide concentration, and weak physiological stability (57), have weakened their development as anti-inflammatory therapeutics. Hybridizing different anti-inflammatory peptides is one of the most successful approaches to obtain a novel anti-inflammatory peptide with increased activity but minimized cytotoxicity (58, 59). Based on previous findings, we designed several hybrid peptides comprising the active center of AMPs, including LL-37 (13-36), YW12D (1-12), IDR-1 (1-13), and CATH2 (1-13), with TP5 or the active center of Tα1 (17-24).

It has been well established that LPS mediates its immune response in macrophages via TLR4 (60). MD-2, an accessory protein of TLR4, is responsible for recognizing LPS; in turn, LPS interacts with the Toll/Interleukin-1 receptor (TIR) domain on TLR4 and induces the inflammatory effects (61, 62). Therefore, targeting MD-2 is an important therapeutic strategy for the attenuation of the inflammatory response (62–64). Initially, molecular docking was used to simply and effectively scan the binding mode of the anti-inflammatory peptides. LTP, LTA, YTP, and CTP were the hybrid peptides selected for further study, and the anti-inflammatory activities of these four hybrid peptides were assessed in RAW264.7 cells. The four hybrid peptides showed higher anti-inflammatory activity than their parental peptides. LTA, the most active peptide, was selected for a comprehensive analysis.

The hybrid peptides designed in our study were aimed to fight inflammatory disorders with reduced cytotoxicity. Because cytotoxicity is often thought to be a bottleneck for the therapeutic use of these peptides, it was important to evaluate toxicity. In this study, the proliferation assays showed that the hybrid peptides had lower cytotoxic than their parental peptides. Presumably, this decreased cytotoxic activity was due to the rational hydrophobicity of these hybrid peptides, which is similar to that described in other studies (59). The low cytotoxic activity at relatively high peptide concentrations combined with modified anti-inflammatory activity is an excellent combination from the parental peptides.

The present study showed that murine models of intestinal inflammation induced by LPS have characteristics similar to human IBD (14, 65), such as weight loss, neutrophil infiltration, histological features of multiple erosions, and inflammatory intestinal mucosal changes, including crypt abscess. However, LTA treatment significantly reverses weight loss and reduce histological damage such as ulceration of the epithelial and decreased villus height to crypt depth ratio, in the LPS-induced intestinal inflammation experimental model. The infiltration of activated neutrophils is one of the most representative histological features observed in intestinal inflammation, because neutrophils generate superoxide anions and other reactive species (66). The infiltration of activated was significantly increased in LPS-treated mice; however, mice pre-treated with LTA did not show the same effect. The activity of MPO is directly proportional to the neutrophil concentration in the inflamed tissue. Therefore, MPO activity is an index of neutrophil infiltration and inflammation (67). Consistent with this, jejunal MPO activity was markedly increased in LPS-treated mice, but pretreatment with LTA significantly reduced this effect. In addition, MPO activity in the LTA-pretreated group was markedly decreased compared to the Tα1 and LL-37 pretreated groups. Apoptosis is one of the ulcerogenic processes associated with intestinal inflammation (68). In this study, TUNEL staining showed that LPS markedly increased the number of apoptotic cells in the jejunal mucosa, while administration of LTA decreased the extent of apoptosis.

Excessive production of inflammatory cytokines, such as TNF-α, which can amplify the inflammatory cascade by triggering the accumulation and activation of leukocytes, is often seen in intestinal inflammation (69). In the present study, we found that pretreatment with LTA reduced the levels of TNF-α, IFN-γ, IL-6, and IL-1β in the jejunum. Moreover, the levels of TNF-α, IFN-γ, IL-6, and IL-1β in LTA-pretreated group were markedly decreased compared to the Tα1- and LL-37-pretreated groups.

Collectively, these results support the deduction that LTA is the most active peptide that prevents LPS-induced impairment in mice. To identify the mechanisms of the observed anti-inflammatory effects in LPS-treated mice, a comprehensive and detailed analysis was conducted.

LPS can upregulate ~100 different genes, including pro-inflammatory cytokines, signaling molecules, and transcriptional regulators; thus, it can induce several functional responses that contribute to immunity (70). LPS has high biological activity and plays an important role in the pathogenesis of intestinal inflammation (14). In this study, we found that LTA-pretreated mice had significantly reduced plasma LPS levels compared to the LPS-only treated. *In vitro* experiments showed that LTA can almost completely neutralize LPS at a concentration of 8 μg/mL. By neutralizing LPS, LTA could significantly attenuate the intestinal inflammatory effects by reducing the binding of LPS to the TLR4 receptor in the immune cells *in vivo*.

The intestinal mucosa forms a physical and metabolic barrier against toxins and pathogens from the lumen into the circulatory system (71). Deterioration of the intestinal epithelial barrier increases host susceptibility to luminal pathogens and antigens, leading to the chronic intestinal immune response (72). This deterioration is also a key contributing factor in the pathogenesis of intestinal inflammation (73). First, we evaluated the effect of LTA on gut epithelial barrier function via the TEER tests and the results showed that LTA alleviated LPS-induced permeability. The intestinal epithelial barrier is formed by an interplay between different types of barrier components, such as intercellular TJ proteins (74). TJs are responsible for limiting the paracellular movement of compounds across the intestinal mucosa (75). Regions of increased permeability in the TJs are major sites for both infections and the initiation of inflammation in the gut (76). Our data indicated that the expression of two major TJ proteins, Occludin and ZO-1, was regulated by LTA. In addition, TEM was used to determine the TJs between gut epithelial cells, and its results support the protective effect of LTA against LPS-induced damage in jejunum tissues. The effect of LTA on the epithelial barrier suggests that LTA could protect the host by preventing toxins and luminal antigens from impairing the body's defense mechanism, thereby reducing the severity of intestine inflammation.

The hybrid peptides proposed in this study antagonize the effects of LPS in RAW264.7 cells and in the mouse by binding to the TLR4-MD2 complex. To identify the binding ability of LTA to the TLR4/MD-2 complex, binding assays were performed by flow cytometry and confocal laser-scanning microscopy. The results demonstrated that that LTA competitively blocks LPS binding to the TLR4/MD-2 complex. Consistently, the MD simulation showed that LTA binds to the hydrophobic pocket of MD-2, which partially overlaps with the LPS binding sites on MD-2 (52). This binding mode could be the cause of the LPS-antagonizing effect. Therefore, the present study indicated that LTA confers its anti-inflammatory activity by blocking LPS binding to the TLR4/MD-2 complex.

NF-κB signaling regulates cytokines and cells involved in the inflammatory process (77), and LPS is a strong activator of NF-κB, through its interactions with TLR4. NF-κB is considered to be a crucial initiative factor regulating inflammatory gene expression (78). Thus, we tested the expression of the major proteins involved in the NF-κB pathway to clarify the anti-inflammatory mechanism of LTA in intestinal inflammation. LTA effectively inhibited the activation of NF-κB signaling by suppressing of phosphorylation of IKK-β, IκB-α, and NF-κB.

## Conclusion

**Figure 13:**
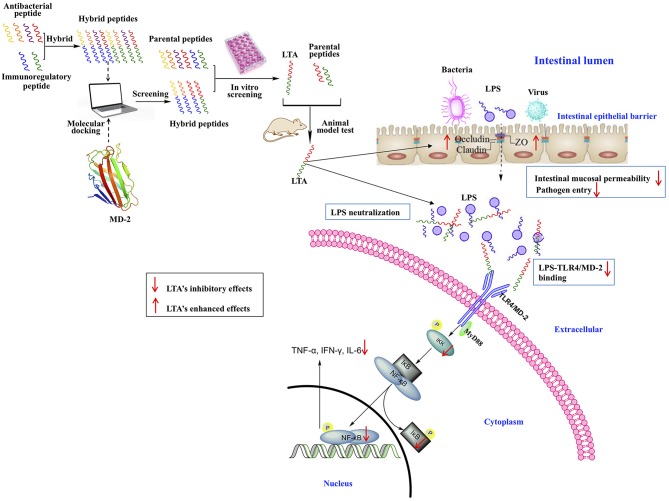
Schematic illustration of design and development of the novel hybrid peptide, LTA for the treatment of intestinal inflammation.

In this study, a feasible approach for the design of anti-inflammatory peptides by the hybridization of different native anti-inflammatory peptides was proposed (Figure 13). The anti-inflammatory potency of the peptides was enhanced while cytotoxicity was reduced. Moreover, the different hybrid peptide combinations may provide a range of opportunities for obtaining a more active anti-inflammatory peptide.

One novel hybrid peptide, LTA, was most effective at reducing the LPS-induced inflammatory response. This peptide, which was identified by molecule docking and *in vitro* experiments, had low cytotoxicity. Our study also confirmed that the anti-inflammatory effects of LTA on the LPS-induced murine intestinal inflammation model may be associated with the neutralization of LPS, the maintenance of the TJ network, the binding activity on the TLR4/MD-2 complex, and the inhibition of the NF-κB signal pathway. Thus, LTA is able to modulate TJ proteins, such as Occludin and ZO-1, and inhibit the production of inflammation mediators, such as TNF-α, IFN-γ, IL-6, and IL-1β. As a result of its broad effects against inflammation, LTA exhibits great potential as a useful tool to study or potentially treat inflammatory disorders.

## Data Availability

All datasets generated for this study are included in the manuscript and/or the supplementary files.

## Ethics Statement

All of the animal experiments were approved by the Institutional Animal Care and Use Committee of China Agricultural University and were performed in accordance with guidelines set forth by the Care and Use of Laboratory Animals of the Ministry of Science and Technology of China (certificate of the Beijing Laboratory Animal employee, ID: 18086).

## Author Contributions

LZ, XW, RZ, JP, and DS conceived the project and designed the experiments. LZ, XW, ZL, JC, and MD conducted experiments. LZ and JP wrote the manuscript and analyzed data. All authors read and commented on the manuscript. The authors are in debt to JP for his contribution toward English proficiency.

### Conflict of Interest Statement

The authors declare that the research was conducted in the absence of any commercial or financial relationships that could be construed as a potential conflict of interest.

## Abbreviations

AMPs: antimicrobial peptides
IDR: innate defense regulator
CATH2: cathelicidin 2
TP5: Thymopentin
Tα1: Thymosin alpha 1
LPS: lipopolysaccharide
LTA: LL-37-Tα1
TNF-α: tumor necrosis factor-alpha
IFN-γ: interferon-gamma
IL-6: interleukin-6
TLR4/MD-2: Toll-like receptor 4/myeloid differentiation factor 2
NF-κB: nuclear factor-kappa B
IBD: inflammatory bowel diseases
UJ: ulcerative jejunitis
TLRs: Toll-like receptors
MAPK: mitogen-activated protein kinase
MyD88: myeloid differentiation primary response 88
LTP: LL-37-TP5
YTP: YW12D-TP5
YTA: YW12D-Tα1
ITP: IDR-1-TP5
ITA: IDR-1-Tα1
CTP: CATH2-TP5
CTA: CATH2-Tα1
DMEM: Dulbecco's modified Eagle's medium
FBS: fetal bovine serum
CCK-8: Cell Counting Kit-8
SPF: specific pathogen free
PMB: Polymyxin B
H&E: hematoxylin-eosin
HRP: horse-radish peroxidase
DAB, 3: 3′diaminobenzidine
DAPI: 4,6-diamidino-2-phenylindole
TUNEL: deoxynucleotidyl transferase mediated dUTP nick end labeling
PPs: Peyer's patches
TEM: Transmission electron microscopy
TJ: tight junctions
TEER: transepithelial electrical resistance
PD: potential difference
Isc: short-circuit current
RT: total electrical resistance
MPO: myeloperoxidase
ZO-1: zonula occluden-1
CE TAL: Chromogenic End-point Tachypleus Amebocyte Lysate
MD: Molecular Dynamics
NPT, number, pressure: and temperature
NVT, number, volume: and temperature
MM-PBSA: Poisson-Boltzmann accessible surface area
IBD: inflammatory bowel diseases.
